# Insidious opioid-induced respiratory depression following abdominal steel pipe perforation injury: A case report

**DOI:** 10.1097/MD.0000000000045435

**Published:** 2025-10-24

**Authors:** Xiaoming Zhang, Shina Qiao, Hongying Pan

**Affiliations:** a Nursing Department, Sir Run Run Shaw Hospital, Zhejiang University School of Medicine, Hangzhou, Zhejiang, China.

**Keywords:** case report, opioid-induced respiratory depression, opioids, perforation injury

## Abstract

**Rationale:**

Opioid-induced respiratory depression (OIRD) is a potentially fatal complication associated with postoperative opioid use, even in low-risk populations. The subtle onset and progression of OIRD can delay detection, potentially leading to cardiorespiratory collapse within minutes.

**Patient concerns:**

A 55-year-old opioid-naïve male who underwent emergency surgery for abdominal penetrating trauma and unstable pelvic fracture. Postoperatively, despite sufentanil-based patient-controlled intravenous analgesia (PCIA), the patient experienced persistent moderate-to-severe pain. After acute pain service adjustment of PCIA parameters, the patient developed sudden unconsciousness with respiratory depression (respiratory rate, 7 breaths/min), hypoxemia (SpO_2_, 90%), bilateral 2-mm pinpoint pupils with sluggish reflexes, and generalized rigidity, despite no additional PCIA activations.

**Diagnoses:**

The critical care team promptly recognized the signs of opioid-induced wooden-chest syndrome, a rare and severe form of OIRD and implemented targeted interventions.

**Interventions:**

Initial administration of naloxone failed to reverse symptoms. The patient required urgent endotracheal intubation, during which marked chest wall rigidity was observed.

**Outcomes:**

These timely interventions enabled the successful rescue of the patient, who was transferred back to a general nursing unit on postoperative day 2.

**Lessons:**

This case of OIRD due to opioid-induced wooden-chest syndrome underscores the danger of omitting dose titration in opioid-naïve patients. We therefore advocate for vigilant monitoring, strict titration protocols, and enhanced staff training to manage such emergencies.

## 1. Introduction

Opioids remain a cornerstone of postoperative pain management. However, their use risks of serious complications including oversedation and respiratory depression.^[1]^ Opioid-induced respiratory depression (OIRD) manifests clinically as bradypnea, which in severe cases leads to respiratory arrest and even cardiopulmonary arrest.^[2]^ A meta-analysis^[3]^ has identified preoperative comorbidities (cardia/pulmonary disease, obstructive sleep apnea [OSA]) as independent risk factors are more susceptible to OIRD. Additionally, the concomitant use of sedatives such as benzodiazepines or gabapentin together with opioids can also increase the incidence of OIRD. Some cases of OIRD progress gradually from somnolence, oversedation, and slowed breathing to respiratory arrest, while others can occur suddenly without any preceding signs. Herein, we report a case of occult OIRD where the patient experienced a sudden onset of OIRD while in a state of persistent moderate-to-severe pain.

## 2. Case presentation

A 55-year-old male presented to the emergency room with a combined lumbar and abdominal penetrating injury caused by a falling steel pipe. His medical history was unremarkable. The patient was 165-cm tall, weighed 70 kg, body mass index 25.7 kg/m², and had no symptoms suggestive of OSA, such as hypertension, snoring, witnessed apnea, or daytime sleepiness. Emergency surgery was performed, comprising exploratory laparotomy, foreign body removal from the abdominal penetrating injury, and open reduction and internal fixation of the pelvic fracture. General anesthesia was induced with sufentanil (Yichang Humanwell Pharmaceutical Co., Ltd., China; 0.4 mcg/kg), propofol (Propoven, Fresenius Kabi Austria GmbH; 1.7 mg/kg), and rocuronium bromide (Esmeron, Siegfried Hameln GmbH, Germany; 0.8 mg/kg). Maintenance consisted of 1.5% sevoflurane (Yian, Shanghai Hengrui Pharmaceutical Co., Ltd.), oxygen (2 L/min), remifentanil (RUIGIE, China National Pharmaceutical Group Industry Co., Ltd. Langfang Branch; 600 mcg/h), propofol (3.5 mg/kg/h), and dexmedetomidine (Aibaining, Jiangsu Hengrui Pharmaceuticals Co., Ltd., China; 0.15 mcg/kg/h). The 200-minute procedure concluded with an initiation of patient-controlled intravenous analgesia (PCIA) containing sufentanil (1 mcg/mL) in a 250-mL solution, programmed with a basal infusion rate of 1 mL/h, bolus dose of 2 mL, 5-minute lockout interval, and 12-mL hourly limit.

The patient was transferred to the intensive care unit (ICU) for mechanical ventilation and continuous respiratory monitoring, with respiratory rate (RR) and pattern documented at 15-minute intervals. At 2 hours postoperatively, the patient regained consciousness with effective spontaneous breathing, intact cough reflex, and Grade 5 muscle strength in both upper extremities. Arterial blood gas analysis demonstrated normal parameters, prompting extubation and initiation of supplemental oxygen via nasal cannula (3 L/min). Continuous respiratory monitoring recorded hourly RR and rhythm. Surgical site pain was rated 5/10 on the 11-point numerical rating scale (NRS).

On postoperative day 1 (POD 1) morning, the acute pain service (APS) team conducted a pain assessment. The PCIA device had delivered 139-mL total volume with 60 activation attempts. The patient reported persistent dull pain exacerbated by movement, with an NRS of 4/10 at rest, and 9/10 during activity. Insufficient analgesia persisted despite device activations. The PCIA bolus dose was consequently increased to 3 mL. Following satisfactory pain control achievement, no opioid-related adverse effects (e.g., nausea, vomiting, dizziness, or altered consciousness) were documented.

One hour following PCIA parameters adjustment, the patient developed sudden-onset unconsciousness accompanied by respiratory depression (RR, 7 breaths/min) and hypoxemia (SpO_2_, 90%). Pupils constricted to 2 mm bilaterally with sluggish light reflex were observed alongside generalized muscular rigidity. The clinical presentation met diagnostic criteria for OIRD. Immediate interventions comprised high-flow oxygen via mask (10 L/min), immediate PCIA discontinuation, and intravenous administration of naloxone 0.4 mg. However, the patient’s SpO_2_ progressively deteriorated to 75%, necessitating emergency endotracheal intubation with subsequent mechanical ventilation (mode: pressure support ventilation; fraction of inspired oxygen [FiO_2_] 50%; tidal volume 380 mL). Significant chest wall rigidity was observed during intubation. Arterial blood gas analysis obtained 10 minutes post-ventilation initiation revealed: pH 7.131, PaCO_2_ 86 mm Hg, HCO_3_^-^ 28.2 mmol/L, and PaO_2_/FiO_2_ ratio 188.

**Figure 1.**
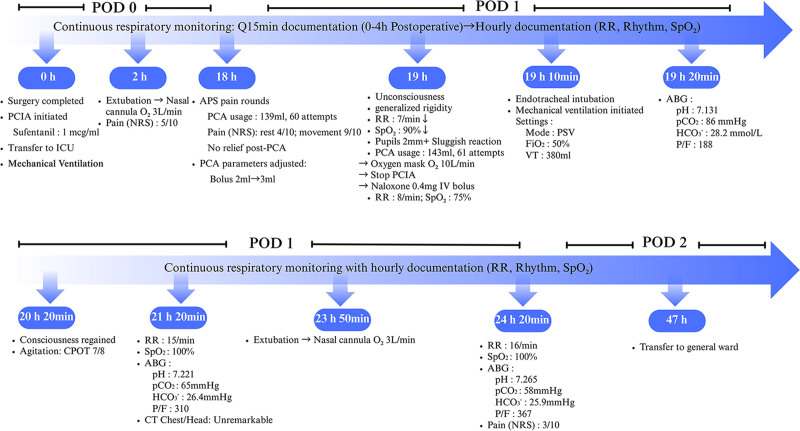
Chronological summary of the patient’s clinical course, including critical timepoints (surgery completion, OIRD onset, and interventions), opioid administration adjustments (PCIA parameters), and physiological markers (RR, SpO₂, pain scores). OIRD = opioid-induced respiratory depression, PCIA = patient-controlled intravenous analgesia, RR = respiratory rate.

One hour after initiating mechanical ventilation, the patient regained consciousness with marked agitation, demonstrated by a Critical Care Pain Observation Tool score of 7/8. Given the confirmed diagnosis of OIRD, the critical care team implemented a conservative analgesic strategy, deliberately avoiding additional opioid administration to prevent further respiratory compromise. Critical review of PCIA records revealed the complete absence of device activations from the time of APS parameter adjustment until RR and SpO_2_ deterioration. Two hours after initiating mechanical ventilation, the patient’s respiratory status stabilized with the following parameters: RR 15 breaths/min, SpO_2_ 100%, pH 7.221, PaCO_2_ 65mmHg, HCO_3_^-^ 26.4 mmol/L, and PaO_2_/FiO_2_ ratio 310. Chest and cranial computed tomography revealed no pathological findings. Four and a half hours post-ventilation initiation, successful extubation was achieved with supplemental oxygen via nasal cannula (3 L/min). Subsequent reassessment 30 minutes post-extubation demonstrated stable respiratory parameters: RR 16 breaths/min, SpO_2_ 100%, pH 7.265, PaCO_2_ 58mmHg, HCO_3_^-^ 25.9mmHg, and PaO_2_/FiO_2_ ratio 367. The patient maintained a pain score of NRS 3 and was transferred to general ward on POD 2. Follow-up assessments on PODs 3, 5, and 7 demonstrated no opioid-related complications including somnolence or oversedation. The chronological progression of clinical events is summarized in Figure 1.

## 3. Discussion

PCIA with opioids is a widely utilized postoperative pain management strategy following major surgery. Although the incidence of OIRD is reported at 1.21%,^[4]^ it constitutes the primary proximate cause of death in opioid overdoses.^[5]^ Most OIRD cases occur within the initial 12 postoperative hours,^[6]^ typically attributable to residual anesthetic effects and intensive opioid administration during early postoperative phase. Notably, this case involved a patient monitored in the ICU for nearly 24 hours postoperatively, resumed oral intake, and demonstrated full anesthetic recovery. Beyond opioid naïvety and PCIA use, no established OIRD risk factors were identified.^[7]^ Paradoxically, the patient exhibited sustained moderate-to-severe pain postoperatively. Given pain’s counter-regulatory effects on respiratory depression via tachypnea induction,^[8]^ this clinical scenario presented an exceptionally low theoretical risk for OIRD development.

The etiology of OIRD is recognized as multifactorial. Hamada et al reported a rare case of respiratory depression secondary to pharmacodynamic interactions between oxycodone and antiemetics.^[9]^ Although the precise mechanism remains undetermined, 2 critical observations emerge: first, unlike opioid titration in cancer pain management, a mandatory safety measure to prevent overdose,^[10]^ postoperative PCIA opioid dosing relies predominantly on an anesthesiologist’s clinical judgment and patient-specific parameters during device configuration. In this case, the opioid-naïve patient received a PCIA bolus dose escalation to 3 mL without prior titration by the APS. Although 1 additional bolus was self-administered within 30 minutes of parameter adjustment, no excessive sedation, significant RR reduction, or oxygen desaturation occurred. However, omitting dose titration when escalating opioids in opioid-naïve patients may precipitate OIRD through rapid drug accumulation exceeding individual tolerance thresholds. Second, the ICU team administered esomeprazole (40-mg IV daily) for stress ulcer prophylaxis, which inhibits hepatic CYP3A4 enzyme activity. This pharmacokinetic interaction potentially impaired sufentanil metabolism, leading to drug accumulation that may have contributed to OIRD.^[11]^ Due to the clinical urgency of restoring adequate ventilation, serum sufentanil concentration measurements were not obtained, precluding confirmation of this hypothesis.

Of particular note, the administration of 0.4-mg naloxone failed to reverse the patient’s condition, which instead continued to deteriorate. This suggested a more complex etiology beyond simple central respiratory depression. A multidisciplinary consultation with intensivists and the APS was convened. We hypothesize that a combination of rapid opioid accumulation in an opioid-naïve patient, potential pharmacokinetic interactions, and ultimately the development of opioid-induced wooden-chest syndrome collectively contributed to the refractory respiratory depression. The constellation of findings, including depressed RR, hypoxia, altered consciousness, and critically, the marked rigidity of the respiratory muscles unequivocally observed during endotracheal intubation, led to a diagnosis of opioid-induced wooden-chest syndrome as the cause of OIRD. This is a severe and distinct manifestation of OIRD, which is notably resistant to standard doses of naloxone.^[12]^

Our institutional safety protocol for opioid PCIA mandates continuous monitoring of vital signs, in general wards for the first 48 hours postoperatively. This is complemented by comprehensive nursing assessment at least every 4 hours, evaluating consciousness, RR, heart rate, blood pressure, SpO_2_, and level of sedation. In the ICU setting, the frequency of these structured assessments is intensified to a minimum of once per hour. It was under this protocol that the ICU nursing staff promptly detected the patient’s initial signs of altered consciousness and respiratory depression. Their vigilant assessment triggered an efficient multidisciplinary response, culminating in effective resuscitation measures that ultimately prevented a fatal outcome. Despite the suddenness and severity of the event, this successful rescue underscores the critical importance of both a structured monitoring protocol and a highly trained clinical team capable of recognizing and managing such emergencies. Furthermore, this case reminds us that in general ward settings, where monitoring intensity is lower, there is likewise a need to enhance surveillance strategies for patients on opioid PCIA while simultaneously strengthening training programs to improve clinicians’ ability to identify and respond to such acute events.

In conclusion, this case provides 2 novel insights that contribute to the understanding of postoperative OIRD. First, it illustrates that opioid-induced wooden-chest syndrome, a severe complication typically associated with anesthetic induction, can occur in the context of postoperative PCIA and is a critical differential diagnosis in cases of naloxone-resistant respiratory depression. Second, it exposes the underappreciated risk of omitting dose titration when escalating PCIA parameters in opioid-naïve patients, demonstrating that rapid opioid accumulation can precipitate catastrophic events even in the absence of traditional risk factors and despite the presence of counter-regulatory pain.

To apply these insights in clinical practice, we recommend: first, enhanced vigilance for atypical presentations of OIRD, such as chest wall rigidity, particularly when naloxone fails. Second, strict adherence to a protocol that mandates slow, incremental dose titration when configuring or adjusting PCIA for opioid-naïve individuals. Third, routine preoperative screening for OSA and other risk factors, with a low threshold for suspecting undiagnosed disease. Finally, implementation of regular, multidisciplinary training programs for healthcare staff to improve the recognition and management of opioid-related emergencies.

## 4. Conclusion

In summary, this case elucidates a rare instance of opioid-induced wooden-chest syndrome causing OIRD in a postoperative PCIA setting and underscores the critical danger of omitting dose titration in opioid-naïve patients. We therefore advocate for enhanced vigilance regarding atypical presentations, strict adherence to gradual titration protocols, and the implementation of risk-stratified monitoring with continuous modalities when feasible, and the establishment of regular multidisciplinary training programs to ensure healthcare staff is proficient in recognizing and managing such opioid-related emergencies.

## Acknowledgments

The authors sincerely thank the patient for permission to report this case.

## Author contributions

**Conceptualization:** Xiaoming Zhang, Shina Qiao, Hongying Pan.

**Data curation:** Xiaoming Zhang.

**Formal analysis:** Xiaoming Zhang, Shina Qiao.

**Funding acquisition:** Xiaoming Zhang.

**Investigation:** Xiaoming Zhang, Shina Qiao.

**Methodology:** Xiaoming Zhang, Hongying Pan.

**Resources:** Hongying Pan.

**Software:** Xiaoming Zhang.

**Supervision:** Hongying Pan.

**Writing – original draft:** Xiaoming Zhang, Shina Qiao.

**Writing – review & editing:** Xiaoming Zhang, Shina Qiao, Hongying Pan.
